# Mental Health and Healthy Habits in University Students: A Comparative Associative Study

**DOI:** 10.3390/ejihpe12020010

**Published:** 2022-01-27

**Authors:** José Antonio Ruiz-Hernández, Ángela Guillén, David Pina, Esteban Puente-López

**Affiliations:** 1 Department of and Psychiatry and Social Psychology, Faculty of Psychology, University of Murcia, 30100 Murcia, Spain; jaruiz@um.es (J.A.R.-H.); angela.guillenj@um.es (Á.G.); 2 Department of Social and Health Sciences, Faculty of Medicine, University of Murcia, 30100 Murcia, Spain; 3 External Service of Forensic Science and Techniques (SECYTEF), University of Murcia, 30100 Murcia, Spain; esteban.puente@um.es

**Keywords:** university students, mental health, healthy habits, area of knowledge, educational level

## Abstract

There is evidence of increased psychopathology in university students and its relationship with unhealthy lifestyle habits. The objective of this work is to examine the prevalence and differences in psychopathological symptoms and lifestyle in a sample of university students according to educational levels and area of knowledge. A comparative associative study was conducted with 1405 university students. The results indicated significant differences in psychopathology and habits in the different groups. The prevalence of psychopathology found was high, especially depressive and anxious pathologies. This incidence tends to be higher in undergraduates and/or Arts and Humanities, coinciding with those who tend to have poorer lifestyle habits. The promising contributions from this study facilitate the early detection of university students with a risk profile for the appearance or exacerbation of psychopathology, as well as the design of psychological intervention programs aimed at the psychological well-being of this population.

## 1. Introduction

Interest in mental health and the well-being of university students has gradually increased in recent decades [1]. The move to university is a stage of constant psychosocial and academic changes, where anxious and depressive symptomatologies usually appear, in addition to the aggravation of previous mental health problems [2,3] Recently, Auerbach et al. [4], in the first phase of the World Health Organization (WHO), Geneva, Switzerland global project for university students, explored the prevalence of psychological disorders in more than 13,000 students from 19 universities in eight countries. In that study, they found that at least 35% of the respondents suffered from mental health problems. The results show that major depression disorder (MDD) was the most common, with a prevalence of 21.2–18.5%, followed by generalized anxiety disorder (GAD) with 18.6–16.7%. In Spain, it was observed that 23.1% of the students showed symptomatology consistent with MDD, and 19.3% with GAD. Moreover, 20% of the students reported comorbidity [2]. The high prevalence of mental health problems found among university students is considered a challenge within the third Sustainable Development Goal (SDG), which seeks to ensure the promotion of all persons at every stage of their lives [5]. For universities, identifying students who could benefit from psychological treatment or preventive actions is a difficult task.

### 1.1. Mental Health According to Educational Level and Area of Knowledge

Although interest in university students’ mental health has been growing in recent years, few studies have examined the relationship between mental health and academic variables such as the current academic year, educational level or area of knowledge to which their degree belongs. Research has focused on undergraduates, mainly freshman students. It has been considered that, when entering university, they face a greater number of vital changes (new social relations, independence, economic responsibility or study methods, among others). Bassols et al. [6] found that 30.8% of freshman students showed symptoms of anxiety compared to 9.4% of senior students. Auerbach et al. [4] found that 83.1% of students with a psychopathology identified in the first academic years had its onset before starting university and that the prevalence of students who develop mental health problems during university years is actually lower. However, others have considered the importance of delving into the mental health problems that occur in the last years of undergraduate or graduate studies associated with academic pressure, stress about future career decisions, competitiveness in the labor market and other age-related responsibilities (family or economic responsibilities) [7,8,9]. For example, the works of Sakellari et al. [7] reported higher state anxiety scores in the last university years, doctoral studies, where a high prevalence of stress and anxiety has been observed [8,9]. The results on the courses or academic levels in which university students show greater emotional problems are inconclusive. No longitudinal studies that could elucidate these data have been found.

The area of knowledge has also been considered a relevant factor in the mental health of university students [10]. Most studies have focused on Health Science students, mainly doctors and nurses, as they are considered a risk sector [11]. Although a high prevalence of stress, anxiety, somatic symptoms and depression has been found in these [6,12,13], some works have found similar or even higher profiles in Engineering, Arts and Humanities, Sciences and/or Social Sciences [7,8,14,15]. Previous studies that delve into the influence of the area of knowledge of their studies do not observe differences when it comes to psychopathology [9]. As is the case with the educational level, the studies that address the differences in mental health according to the area of knowledge are scarce and recent, so more evidence is still needed in this regard. The results are expected to point to what the literature has shown most interest in, i.e., that undergraduate and Health Science students will exhibit high rates of psychopathology.

### 1.2. Healthy Habits According to Educational Level and Area of Knowledge

Healthy lifestyle habits are commonly understood as the incorporation of habits related to a balanced diet, playing sports regularly, rest, hygiene, responsible alcohol consumption and avoiding drug use [5,13,16,17]. In addition to improving physical health, a healthy lifestyle affects people’s emotional well-being, both in the general adult and university populations [13,16]. This relationship suggests the importance of influencing the maintenance of good habits for the promotion of mental health. In Spain, the Healthy Universities Network (REUS) was created with the aim of turning the university into an environment that promotes health and physical, psychological and social well-being [17]. Accordingly, the Healthy University Project was created in a Spanish university based on a cross-sectional study with 956 undergraduate university students from different areas of knowledge. The data obtained showed a high percentage of students who do not eat enough fruits and vegetables daily, almost one out of four students do not exercise, most students drink alcohol regularly but only lightly, while 10% drink every day, and finally, almost 30% smoke [17].

In this sense, it is internationally recognized that university students tend to have unhealthy lifestyle habits due to changes in environment, schedules and freedom of choice for their own lifestyle, such as high consumption of tobacco, drugs and alcohol [8,16], low sports activity [18,19] and/or poor quality and/or few hours of sleep [14,20]. These studies are generally descriptive, and whether these habits relate to the course of study (degree, master’s degree or doctorate) or area of knowledge is unknown. Often, studies on lifestyle habits in university students tend to describe health-related behaviors and relate them to mental health. Whatnall et al. [21], studied the habits of a sample of 3529 undergraduate and postgraduate university students from six areas of knowledge, finding that 89.5% did not consume enough fruit and vegetables, 50.3% drank more than two alcoholic beverages a day, 38.1% performed less than 150 min of physical activity per week and 26% did not sleep between 7–9 h a day. In addition, 37.6% scored high in psychological distress on the Kessler Psychological Distress Scale (K-10) [22]. These authors showed that poor lifestyle habits can affect academic performance and mental health [21]. Likewise, Hoying et al. [13], in a sample of Health Science students, found negative correlations between a healthy lifestyle and mental health measures such as anxiety, depression and stress. Deb et al. [23] revealed that Social Sciences students and Arts and Humanities students suffered more depressive symptomatology than Science students, associating regular exercise with an improvement in mental health, among other variables. Among the few comparative studies are the results of Izsáj et al. [14], who, when comparing Art students with the rest, found that they used substances more frequently and scored significantly higher in hostile and anxious symptomatology. As a hypothesis, this study suggests that students with higher scores in psychopathology will have worse life habits.

### 1.3. Objectives

In recent literature, the mental health of university students has become a focus of interest. Given the evidence that university students tend to present psychopathological pictures and/or symptoms and unhealthy lifestyle habits, this publication aims to go further by exploring their relationships with academic variables related to educational level (degree, master’s and doctoral degrees) and the area of knowledge of Bachelor of Arts or Science. Studies usually examine in depth the students’ mental health in relation to their life habits [8,16] or examine the differences according to either educational level [6,9] or, on the other hand, the area of knowledge [7,15]. However, no studies similar to this one have been found, where all these variables together are considered, especially academic variables. These variables are new and not yet sufficiently explored to draw precise conclusions about their relationship with mental health and university students’ habits.

This study would help to define, with greater precision, the profile of at-risk students for early detection and effective psychological intervention by university health services, especially considering that at least one-fifth of the students with psychological problems apply for therapeutic help [2,10,24].

The main objective of this work is to explore and deepen the study of psychopathological symptomatology and lifestyle in a sample of university students, considering their current educational level and area of knowledge. The following specific objectives are discussed:

1. Prevalence of psychopathological symptomatology in a sample of university students.
2. Differences in psychopathological symptomatology according to area of knowledge and educational level.
3. Differences in lifestyle habits according to educational level and area of knowledge.

## 2. Materials and Methods

### 2.1. Participants

The initial sample included 1476 university students. After applying the exclusion criteria, the final sample included 1405 cases (996 women and 409 men). Participants’ average age was 22.76 years (SD = 4.9), with a range of 18–49 years, and mostly Spanish nationality (95.4%). The students belonged to 3 educational levels: 80.8% were undergraduate students, 10.7% were master’s students and 8.5% were doctoral students. University degrees were grouped according to the Royal Decree 1393/2007 of 29 October, which establishes the official university teachings in 5 areas of knowledge. The basic subjects for each area of knowledge are: Arts and Humanities (Anthropology, Art, Ethics, Artistic Expression, Philosophy, Geography, History, Modern Language, Language, Classical Language, Linguistics, Literature, Sociology), Sciences (Biology, Physics, Geology, Mathematics, Chemistry), Health Sciences (Animal Anatomy, Human Anatomy, Biology, Biochemistry, Statistics, Physics, Physiology, Psychology), Social and Legal Sciences (Anthropology, Political Science, Statistics, Physics, Physiology, Psychology), Social and Legal Sciences (Anthropology, Political Science, Communication, Law, Economics, Education, Business, Statistics, Geography, History, Psychology, Sociology) and Engineering (Graphic Expression, Physics, Computer Science, Mathematics, Chemistry). The students belonged: 14.5% to Arts and Humanities, 15.6% to Sciences, 26% to Health Sciences, 37.7% to Social and Juridical Sciences and 6.2% to Engineering. Of the respondents, 57.2% had a scholarship from the Ministry of Education.

### 2.2. Procedure

This research was conducted at a public University in Spain once it was approved by the Ethics Committee of the University (ID: 2337—2019). Following the classification system of Ato et al. [25], an empirical study following an associative, comparative and cross-sectional design was conducted.

Data collection was preceded by contact with the author of the instrument used for authorization to use it. An evaluation protocol was created that required about 10 min to complete. Then, after receiving the approval of the institution’s ethics committee, the evaluation protocol was designed. The institutional online survey platform (https://www.encuestas.um.es, accessed on 1 July 2019) was used for its dissemination. On the first page, information was provided about the objectives of the study, participation anonymity, data processing and informed consent, which was necessary to continue to complete the survey. The evaluation protocol was sent 5 times by the institutional email to 100% of the university students enrolled in the 2018/2019 academic year. Emails were sent between February and May 2019. Participation was voluntary. Students did not receive any compensation for participating. Inclusion criteria were: (a) to be enrolled in any course and area of knowledge of the university and (b) to accept the informed consent and data processing. Excluded cases were: (a) those that did not complete 100% of the evaluation protocol, (b) cases that showed outliers or random response patterns and/or (c) not being a student of the University of Murcia, Murcia, Spain.

### 2.3. Measures

The evaluation protocol consisted of 43 items with dichotomic and multiple-choice questions. Data on sociodemographic, academic, healthy habits and psychopathological symptomatology were collected. Concerning the sociodemographic variables, personal data such as age, sex, nationality and academic data such as the degree, educational level and having a scholarship from the Ministry of Education were collected.

Healthy habits were evaluated with ad hoc designed items. The weekly frequency of sports performance was evaluated (1 = practically none, 5 = 10 times or more per week), frequency of consumption of alcoholic beverages (1 = never, 5 = 4 or more times per week), frequency of cigarettes smoked per day (1 = I do not smoke, 5 = <40 cigarettes) and daily hours of sleep (1 = less than 6 h, 5 = more than 9 h).

The Symptom Assessment–45 Questionnaire (SA–45) [26], adapted into Spanish by Sandín et al. [27], was used to evaluate psychopathology. Its original version consists of 9 scales with 45 items rated on a 5-point response format (0 = not at all, 4 = very much). Following the results of previous studies [4], in the present study, only the depression scales (in the Spanish adaptation α = 0.85, in our study α = 0.83), anxiety (in the Spanish adaptation α = 0.84, in our study α = 0.84), interpersonal sensitivity (in the Spanish adaptation α = 0.84, in our study α = 0.82), somatization (in the Spanish adaptation α = 0.80, in our study α = 0.77) and hostility (in the Spanish adaptation α = 0.83, in our study α = 0.74) were used.

### 2.4. Data Analyses

Firstly, the variables of interest were generated: dimensions of psychopathological symptomatology, area of knowledge and educational level. From the descriptors, the extreme scores and histograms of each of the variables were examined to detect possible outliers. Exclusion criteria were applied, eliminating 8 cases that did not complete the evaluation protocol and 63 cases with outliers (≤4 and ≥12 h of sleep, ≤18 and ≥50 years and extreme scores in psychopathology).

To describe the sample, we included the means and standard deviations of the continuous variables (sociodemographic and psychopathological symptomatology) and the frequencies and percentages of the categorical variables (academic variables and healthy habits). Regarding psychopathology, reliability of the general scale and the subscales of the instrument used was calculated. To facilitate its description, symptoms were grouped by severity levels (mild, moderate and severe), taking into account the cut-points (mean and standard deviation) proposed by Sandín et al. [27] in a similar sample of university students.

Subsequently, the Kolmogorov–Smirnov statistic together with the Levene test indicated compliance with the assumptions of normality and homoscedasticity of the variables. The ANOVA test was used to calculate the differences between psychopathology and the academic variables, with their respective post-hoc tests (Tukey and Games–Howell). The counterfactual tests allowed us to interpret the intergroup differences. For the interpretation of the magnitude of the effect, the partial eta squared was obtained, understanding a small (*η_p_*^2^ = 0.01), medium (*η_p_*^2^ = 0.06) and large (*η_p_*^2^ = 0.14) magnitude, and Cohen’s *d* [28], understanding a small (*d* = 0.25), medium (*d* = 0.50) and large (*d* = 0.80) magnitude.

Finally, the chi-square test was used to determine the differences between habits and academic variables. All the analyses were performed with the SPSS 26 statistical package.

## 3. Results

### 3.1. Prevalence of Psychopathological Symptomatology in a Sample of University Students

The university students in this study had a high prevalence of clinical symptoms. Table 1 shows that 33.7% presented severe levels in depression, 10.2% in hostility, 17.6% in interpersonal sensitivity, 20.4% in somatization and 45.1% in anxiety. In contrast, only 2.4% presented mild symptoms in depression, 13.4% in somatic symptoms and 17.3% in interpersonal sensitivity.

### 3.2. Differences in Psychopathological Symptomatology According to Educational Levels and Area of Knowledge

After examining the differences in psychopathological symptomatology depending on the educational levels being studied, we observed that, in depression, *F*(2,1402) = 7.45, *p* = 0.001, in interpersonal sensitivity, *F*(2,1402) = 8.53, *p* = 0.00 and in anxiety, *F*(2,1402) = 3.97, *p* = 0.019, there were significantly higher means in the degree compared to the doctorate (see Table 2). In particular, we observed that the prevalence of severe symptomatology in the degree was between 15.5–35.8% versus 0.6–2.8% in the doctorate level. Differences were only observed between the degree, the master’s degree and the doctorate in the variable hostility, with higher means in the degree, *F*(2,1402) = 7.25, *p* = 0.001. The effect size of the significant differences was medium, ranging from 0.24–0.38. No significant differences in somatization were observed.

Depending on the areas of knowledge, significant differences in depression were observed, with a significantly higher mean in Sciences and Arts and Humanities compared with Health Sciences and Social Sciences, *F*(4,1400) = 10.82, *p* = 0.000. Effect sizes in the comparisons of these groups ranged from 0.13–0.45 (see Table 3), with the largest effects found in the differences in Arts and Humanities compared with Health Sciences (*d* = 0.45) and Social Sciences (*d* = 0.40). The group of Health Sciences, with lower means, and the Arts and Humanities group, with higher means, differed from the Engineering group with mild and moderate effect sizes, respectively. In interpersonal sensitivity, the differences were found in Arts and Humanities, with a higher mean than all the others, *F*(4,1400) = 7.74, *p* = 0.000, and a medium-large effect size (*d* = 0.32–0.43). On the contrary, in somatization, the Arts and Humanities group presented a lower mean than the other groups, differing mainly from the Engineering group according to the post hoc test, *F(*4,1400) = 3.27, *p* =0.011. The effect size was small (*d* = 0.02). In anxiety, the Engineering group had a significantly lower mean than the Sciences and Social Sciences groups, whereas the Arts and Humanities group had a significantly higher mean than the other groups, *F*(4,1400) = 9.35, *p* = 0.000. The effect size of the differences in anxiety was medium-large (*d* = 0.36–0.65). No significant differences were observed in hostility (see Table 4).

### 3.3. Differences in Lifestyle Habits According to Educational Levels and Area of Knowledge

Regarding healthy habits, 43.8% of the students played almost no sports, 13.9% drank alcohol more than 2–3 times a week, 14.7% were smokers and 63.3% slept less than 7 h a day.

The results of examining the relationships between habits and the educational levels are presented in Table 5. Significant differences were found in the frequency with which the students played sports, *χ*^2^ = 16.92, *p* = 0.03, and in the consumption of alcoholic beverages, *χ2* = 18.70, *p* = 0.01. On the one hand, we observed that the degree students practiced less sport, that is, 46.4% almost never played sports versus the master’s and doctorate degrees, in which 45.1 and 45.4%, respectively, played sports more than 2 h per week. On the other hand, the master’s degree students reported higher alcohol consumption, as 20.5% reported that they drank with a weekly frequency of more than 2–3 times, whereas 27% of doctoral students never drank alcohol.

Considering the areas of knowledge, significant differences were also found in the sport performed, *χ2* = 28.29, *p* = 0.02 (see Table 6). The lowest frequencies of sports were observed in students of Arts and Humanities and Social Sciences: 47.5% and 46.9%, respectively, hardly practiced at all. The Engineering and Health Sciences groups played more sports than the rest of the groups, reaching 47% of students who exercised more than 2 h per week. Finally, although there were no significant differences, there was a tendency of the Engineering group to consume alcoholic beverages and tobacco less frequently, and 94.3% did not smoke. However, the Arts and Humanities group reported that 18.6% consumed alcohol more than four times per week compared to the mean of 12.9% of the other groups.

## 4. Discussion

This study proposed, firstly, to determine the psychopathological profile of a sample of university students and then to examine the significant differences in psychopathological symptomatology and lifestyle habits according to the area of knowledge and educational levels.

Concerning the first objective, we observed that university students had a high prevalence of moderate–severe psychopathological symptomatology, in line with recent results from other works [29]. Degree students have been identified in the literature as a risk group for presenting high rates of symptoms and/or anxious-depressive pictures, albeit with lower percentages than those found in this study [2,4]. Mood disorders and anxiety are known to occur more frequently in women [3,8]. These data could explain the high prevalence found in our results, as 70.9% of our sample are women and most of them are degree students (80.8%). In any case, it is very important to take into account the possible existence of a self-selection bias, given the methodology used. This phenomenon has been observed in works such as those of Bantjes et al. [3] and Whatnall et al. [21].

Studying the differences between symptomatology and academic variables, a higher incidence of clinical symptoms was observed in degree students than in the master’s and doctoral students. These results support that the literature focuses primarily on undergraduate students [3,4,13]. However, the effect sizes observed in the comparisons were medium, so the results should be interpreted with caution. Degree students were found to differ mainly from doctoral students in depressive symptomatology and interpersonal and anxious sensitivity, reaching severe levels of up to 30%. These high numbers may be due to the screening scale used, but other works that used criteria from the *Diagnostic and Statistical Manual of Mental Disorders* [30] also observed this trend, even if the figures were lower [31]. Despite the fact that recent research alerts about the mental health of doctoral students when comparing them to the general population [32] and groups with higher education such as high school, degree or master’s degree, they used a different methodology than in our study [9]. In order to reach a consensus, it would be advisable to carry out comparative studies between undergraduates and graduates using the same methodology. These latter authors, deepening the study of conditions that improve or worsen the postgraduates’ mental health, found that interest in the academic career and a positive perception of the future work are protective factors [4,9].

Second, the differences in psychopathology between the different academic areas were analyzed. Although the literature has focused on university students of Health Sciences due to the high prevalence of anxious-depressive symptomatology, this study found that the students of Arts and Humanities present higher rates of depressive, anxious and interpersonal sensitivity symptomatology. This could be due, among other aspects, to the higher percentage of women enrolled in this discipline [33] and the known high prevalence of mental health problems in their case. Despite the limited work in this line, we observed similar results. Thus, for example, Lipson et al. [10], in a sample of 64,519 students from 81 universities, found that students of Arts and Humanities had higher scores in psychopathology indicators than the other areas. The same happens with the results obtained by Deb et al. [23], who found that students of Arts and Humanities together with those of Social and Legal Sciences experienced a higher prevalence of symptomatology, specifically depressive, than Science students. In turn, Iszáj et al. [14], when comparing Arts students with a heterogeneous set of students from other areas, found a greater predisposition in the former to develop psychopathological symptoms. It is suggested that this may be related to the specific stressors they face, linked to the pressure towards creativity, innovation and originality [10,14].

Martínez et al. [34] and Morales et al. [24] noted that students committed to their studies tend to have more psychological resources and this, in turn, improves their mental health and academic performance. The relationship between mental health and academic performance has been well established [1]. A low university entrance score and a high dropout rate may suggest difficulties in academic performance and, consequently, poor mental health. In this sense, the figures of the Spanish university system of the 2018/19 academic year indicate that both the lowest access grade for degree studies and the highest dropout rate after the first year of the year coincide with the studies of Arts and Humanities, as opposed to the studies of Health Sciences [33]. Although the literature has focused mainly on health disciplines, these academic indicators and their relationship to psychopathology should alert students of Arts and Humanities. As an explanatory hypothesis, these students could be considered to have previous anxious-depressive problems that reduced their academic grade and, therefore, the possibility of choosing the desired university degree or a career with high academic demand such as Health Sciences. Although high prevalences of psychopathology were found in Health Sciences students, some authors have considered them as having a greater perception of warning signs, resources for their management and greater willingness to seek therapeutic help compared with the degrees of Science, Social Sciences and Engineering [8,10,35]. In this study, Health Science students showed low rates of depressive symptomatology, suggesting that academic engagement—prior and during university studies—and knowledge about health and disease could act as protective factors for the prevention and promotion of mental health.

Concerning the third objective, there is no consensus in the literature as to which student profile tends to have the worst lifestyle habits. Nevertheless, there is evidence that bad habits, such as low sports activity, poor sleep quality and substance use are among the factors related to the mental health of university students [12,13,21]. Along these lines, on the one hand, the majority of university students in this study do not meet the general recommendations for healthy lifestyles recommended by the WHO, Geneva, Switzerland [5]. These data point in the same direction as those of the last cross-sectional study carried out at the same university [17]. On the other hand, the students in our sample who have the most clinical symptoms are the ones who present the least healthy behaviors, and vice versa. In this sense, it is observed that undergraduate and Arts and Humanities students played less sport and present the highest prevalence of psychopathological symptomatology. In contrast, Engineering students report fewer clinical symptoms and more healthy practices. Of the habits contemplated, sports have shown the clearest results. Works such as those of Cecchini et al. [36] and Whatnall et al. [21] have found that moderate and high levels of physical activity were significantly and inversely associated with anxiety and depression. In this sense, sports could act as a protective factor of mental health. Regarding alcohol consumption, there is a trend for undergraduate and master’s students to consume more alcoholic beverages and tobacco than doctoral students. In the literature, the consumption of alcohol and/or substances have been considered strategies to deal with depressive symptoms and stress [18,37].

### 4.1. Strengths and Limitations

The main strength of this work is the theoretical and empirical contribution of academic variables, still little studied, to the knowledge of mental health in university students. Literature on undergraduate studies is abundant, and there is agreement about the existence of poor mental health, but the studies are descriptive and focus mostly on the first course or knowledge areas such as Health Sciences. To date, no studies have been found that take into account all these variables together. In other words, studies do not consider university students’ mental health and life habits according to the educational level and area of knowledge of their degree.

Despite these strengths, this work is not without limitations. On the one hand, the design used does not allow us to make causal inferences. On the other hand, having a self-selected sample and self-reporting measures may have influenced the high prevalence of symptoms found. Regarding habits, sleep has been considered a significant indicator of psychopathology, but a characteristic trend was not found in this study. Ghrouz et al. [20] measured the quality of sleep and found an association with health problems. As in this study, Whatnall et al. [21] measured sleep as the number of hours, finding results similar to ours.

### 4.2. Implications for Future Research

In future work, it would be interesting to consider the above-mentioned aspects about the methodology used and the proposal to carry out randomized studies, which would allow for precisely determining the relationship between academic variables and mental health.

To detect and intervene psychologically at early stages, it would be interesting to explore psychopathology and habits throughout each of the university years, also taking into account the possible stressors and specific coping in each course and discipline.

### 4.3. Implications for Practice

The results of this study suggest paying attention to non-health disciplines such as Arts and Humanities. In this sense, non-health disciplines and older students have been considered to have more negative attitudes towards seeking help due, among other reasons, to the low awareness of the benefits of these services [10]. The psychological care incorporated in universities has a major role in the detection and early intervention on difficulties and/or psychological problems of the student body. This information is mainly useful for them. The design of a psychological intervention program that has as its general objective the psychological well-being and healthy habits of the participants, together with an active search for students with a risk profile as described in this study, would improve, in addition to their mental health, the academic performance and employability of future graduates. These actions specifically contribute to the third Sustainable Development Goal related to health. Guidance designed to contribute to achieving the SDGs in universities, specifically health, advocates among other measures: facilitating access to care services on campus, providing programs for student wellness, implementing “no smoking” policies on campus and ensuring practices to avoid alcohol and drug use [38].

## 5. Conclusions

The prevalence of psychopathology and unhealthy lifestyle habits is high in university students. There are significant differences with respect to academic variables in both psychopathology (depression, interpersonal sensitivity and anxiety) and sports practice. The literature finds that there is an inverse relationship between these constructs. In this work, undergraduate students presented higher scores of psychopathological symptoms than doctoral students. At the same time, these are the ones who practice sports less frequently and tend to have less healthy practices. Similar results were found for students of Arts and Humanities, according to the branches of knowledge. Early detection of risk profiles would facilitate prevention and psychological intervention on these high prevalences.

## Figures and Tables

**Table 1:** Frequency and prevalence of symptomatology by severity levels.

Symptomatology (SA–45) ^1^	*M* ± *SD*	Mild *	Moderate *	Severe *
Depression	7.6 ± 4.8	39 (2.4)	893 (63.6)	473 (33.7)
Hostility	2.5 ± 2.8	-	1261 (89.8)	144 (10.2)
Interpersonal Sensitivity	5.9 ± 4.5	244 (17.3)	914 (65.1)	247 (17.6)
Somatization	4.4± 3.7	189 (13.4)	930 (66.2)	286 (20.4)
Anxiety	6.9 ± 4.2	-	814 (57.9)	591 (45.1)

* Data were presented as *n* (%). ^1^ Symptom Assessment–45 Questionnaire.

**Table 2:** Psychopathological symptomatology (SA–45) according to the educational levels.

SA–45	Degree (Degr) *	Master’s Degree (M) *	Doctorate (PhD) *	*F*_(2,1402)_	*η_p_* ^2 6^	*d* ^7^ (95% CI)	Post Hoc
D ^1^	7.86 ± 4.83	7.18 ± 4.50	6.18 ± 4.53	7.45 ***	0.01	0.35 (0.16, 0.54)	PhD-Degr ^8^
H ^2^	2.68 ± 2.89	1.98 ± 2.56	1.92 ± 2.46	7.25 ***	0.01	0.25–0.27 (0.08, 0.46)	PhDM-Degr ^9^
SI ^3^	6.16 ± 4.61	5.48 ± 4.30	4.46 ± 3.67	8.53 ***	0.01	0.38 (0.19, 0.56)	PhD-Degr ^9^
S ^4^	4.48 ± 3.76	4.59 ± 3.74	4.45 ± 3.89	0.06	-	-	-
A ^5^	7.14 ± 4.35	6.58 ± 3.97	6.10 ± 4.17	3.97 **	0.005	0.24 (0.05, 0.43)	PhD-Degr ^9^

* Data were presented as mean ± standard deviations. ^1^ Depression; ^2^ hostility; ^3^ interpersonal sensitivity; ^4^ somatization; ^5^ anxiety; ^6^ partial eta squared; ^7^ range of the effect size of significant comparisons; ^8^ Tukey test, ^9^ Games–Howell; ** *p* ≤ 0.05. *** *p* ≤ 0.001.

**Table 3:** Effect sizes of significant SA–45 comparisons and area of study.

SA–45	*d*^6^ (95% CI)	*d*^7^ (95% CI)	*d*^8^ (95% CI)	*d*^9^ (95% CI)	*d*^10^ (95% CI)	*d*^11^ (95% CI)	*d*^12^ (95% CI)	*d*^13^ (95% CI)	*d* ^14^ (95% CI)	*d*^15^ (95% CI)
D ^1^	0.35 (0.18, 0.52)		0.30 (0.14, 0.45)		0.45 (0.28, 0.63)	0.13 (−0.12, 0.38)	0.40 (0.24, 0.56)		0.32 (0.08, 0.55)	
H ^2^						-	-			
SI ^3^					0.40 (0.23, 0.57)	0.32 (0.07, 0.57)	0.43 (0.26, 0.59)			
S ^4^						0.02 (−0.23, 0.27)				
A ^5^		0.43 (0.17, 0.68)			0.41 (0.24, 0.58)	0.65 (0.39, 0.90)	0.36 (0.20, 0.52)			0.30 (0.07, 0.53)

^1^ Depression; ^2^ hostility; ^3^ interpersonal sensitivity; ^4^ somatization; ^5^ anxiety; ^6^ effect size of the Sciences and Health Sciences comparison; ^7^ effect size of the Sciences and Engineering comparison; ^8^ effect size of the Sciences and Social Sciences comparison; ^9^ effect size of the comparison of Arts and Humanities and Sciences; ^10^ effect size of the comparison of Arts and Humanities and Health Sciences; ^11^ effect size of the comparison of Arts and Humanities and Engineering; ^12^ effect size of the comparison of Arts and Humanities and Social Sciences; ^13^ effect size of the comparison of Social Sciences and Health Sciences; ^14^ effect size of the comparison of Engineering and Health Sciences; ^15^ effect size of the comparison of Engineering and Social Sciences.

**Table 4:** Psychopathological symptomatology (SA–45) according to the area of study.

SA–45	Sciences (A) *	Health Sciences (B) *	Engineering (C) *	Social Sciences (D) *	Arts and Humanities (E) *	*F*_(4,1400)_	* η_p_* ^2 6^	Post Hoc
D ^1^	8.53 ± 4.72	6.85 ± 4.80	8.40 ± 5.18	7.17 ± 4.56	9.04 ± 4.86	10.82 ***	0.02	BD-AE ^7^; B-C-E ^7^
H ^2^	2.25 ± 2.72	2.53 ± 2.92	2.16 ± 2.60	2.60 ± 2.72	2.91 ± 3.13	1.89	0	-
IS ^3^	6.37 ± 4.68	5.56 ± 4.49	5.90 ± 4.53	5.49 ± 4.34	7.38 ± 4.63	7.74 ***	0.02	BDC-E ^7^
S ^4^	4.30 ± 3.61	4.27 ± 3.67	3.85 ± 3.47	4.30 ± 4.53	3.77 ± 4.09	3.27 **	0	C-E ^7^
A ^5^	7.25 ± 4.09	6.66 ± 4.07	5.52 ± 3.99	6.82 ± 4.28	8.41 ± 4.64	9.35 ***	0.02	CB-E ^7^; C-DA ^7^; D-E ^7^

* Data were presented as mean ± standard deviations. ** *p* ≤ 0.05, *** *p* ≤ 0.001; ^1^ depression; ^2^ hostility; ^3^ interpersonal sensitivity; ^4^ somatization; ^5^ anxiety; ^6^ partial eta squared; ^7^ Tukey test.

**Table 5:** Healthy habits according to the educational levels.

	Degree *	Master’s Degree *	Doctorate *	*χ* ^2^	df	*p*
Sport				16.92	8	0.03
Practically nothing	526 (46.4)	52 (34.4)	38 (31.9)			
1–2 h/week	186 (16.4)	31 (20.5)	27 (22.7)			
2–5 h/week	283 (24.9)	47 (31.2)	38 (31.9)			
5–10 h/week	107 (9.4)	18 (11.9)	12 (10.1)			
≥10/week	33 (2.9)	3 (2.0)	4 (3.4)			
Alcohol				18.7	18	0.01
Never	191 (16.8)	29 (19.2)	32 (27)			
1 month	370 (32.6)	43 (28.5)	26 (21.8)			
2–4/month	429 (37.8)	48 (31.8)	43 (36.1)			
2–3/week	124 (10.9)	28 (18.5)	16 (13.4)			
≥4/week	21 (1.9)	3 (2.0)	2 (1.7)			
Smoking				2.57	6	0.86
I do not smoke	962 (84.8)	130 (86.1)	106 (89.1)			
<10 cigarettes	120 (10.6)	14 (9.3)	10 (8.4)			
10–20 cigarettes	49 (4.3)	6 (4.0)	3 (2.5)			
≥20 cigarettes	4 (0.3)	1 (0.6)	0 (0)			
Sleep				11.82	8	0.15
≤6 h	280 (24.8)	25 (16.6)	24 (20.2)			
6–7 h	435 (38.3)	74 (49.0)	52 (43.7)			
7–8 h	328 (28.7)	44 (29.1)	35 (29.4)			
8–9 h	71 (6.3)	8 (5.3)	6 (5.0)			
≥9 h	21 (1.9)	0 (0)	2 (1.7)			

* Data were presented as *n* (%).

**Table 6:** Lifestyle habits according to area of study.

	Sciences *	Health Sciences *	Engineering *	Social Sciences *	Arts and Humanities *	*χ* ^2^	df	*p*
Sports						28.29	16	0.02
Practically nothing	92 (42.0)	149 (40.7)	30 (34.5)	248 (46.9)	97 (47.5)			
1–2 h/week	50 (22.8)	67 (18.3)	16 (18.4)	74 (14.0)	37 (18.1)			
2–5 h/week	50 (22.8)	105 (28.7)	29 (33.3)	131 (24.8)	53 (26.0)			
5–10 h/week	21 (9.6)	29 (7.9)	9 (10.3)	61 (11.5)	17 (8.3)			
≥10 h/week	6 (2.7)	16 (4.4)	3 (3.4)	15 (2.8)	0 (0)			
Alcohol						24.7	16	0.07
Never	34 (15.5)	63 (17.2)	20 (23.0)	95 (18.0)	40 (19.6)			
1/month	83 (37.9)	117 (32.0)	27 (31.0)	149 (28.2)	63 (30.9)			
2–4/month	63 (32.9)	142 (38.8)	29 (33.3)	214 (40.5)	63 (30.9)			
2–3/week	29 (11.4)	42 (11.5)	10 (11.5)	62 (11.7)	29 (14.2)			
≥4/week	9 (2.3)	2 (0.5)	1 (1.1)	9 (1.7)	9 (4.4)			
Smoking						19.89	12	0.06
I do not smoke	197 (90.0)	315 (86.1)	82 (94.3)	435 (82.2)	169 (82.8)			
<10 cigarettes	15 (6.8)	36 (9.8)	4 (4.6)	61 (11.5)	28 (13.7)			
10–20 cigarettes	7 (3.2)	14 (3.8)	1 (1.1)	29 (5.5)	7 (3.4)			
≥20 cigarettes	0 (0)	1 (0.3)	0 (0)	4 (0.8)	0 (0)			
Sleep						18.79	16	0.28
≤6 h	52 (23.7)	91 (24.9)	26 (29.9)	116 (21.9)	44 (21.6)			
6–7 h	96 (43.8)	156 (42.6)	27 (31.0)	203 (38.4)	79 (38.7)			
7–8 h	61 (2.7)	96 (26.2)	28 (32.2)	163 (30.8)	59 (28.9)			
8–9 h	6 (27.9)	19 (5.2)	6 (6.9)	36 (6.8)	18 (8.8)			
≥9 h	4 (1.8)	4 (1.1)	0 (0)	11 (2.1)	4 (2.0)			

* Data were presented as *n* (%).

## Data Availability

Data presented in this manuscript are available upon request from the corresponding author.
